# Children and Caregivers’ Exposure to Adverse Childhood Experiences (ACES): Association with Children’s and Caregivers’ Psychological Outcomes in a Therapeutic Preschool Program

**DOI:** 10.3390/ijerph15040646

**Published:** 2018-03-31

**Authors:** Yair Ziv, Inbar Sofri, Kristen L. Capps Umphlet, Stephanie Olarte, Jimmy Venza

**Affiliations:** 1 Department of Counseling and Human Development, University of Haifa, Haifa 3498838, Israel; inbarsofri@gmail.com; 2 The Lourie Center for Children’s Social and Emotional Wellness, Rockville, MD 20852, USA; KCapps@louriecenter.org (K.L.C.U.); SOlarte@louriecenter.org (S.O.); JVenza@louriecenter.org (J.V.)

**Keywords:** adverse childhood experiences, social information processing, behavior problems, preschool, social skills, externalizing problems, trauma

## Abstract

Exposure to adverse childhood experiences (ACE) has been found to have a profound negative impact on multiple child outcomes, including academic achievement, social cognition patterns, and behavioral adjustment. However, these links have yet to be examined in preschool children that are already experiencing behavior or social-emotional problems. Thus, the present study examined the links between the caregiver’s and the child’s exposure to ACE and multiple child and caregiver’s outcomes in a sample of 30 preschool children enrolled in a Therapeutic Nursery Program (TNP). Children are typically referred to this TNP due to significant delays in their social emotional development that often result in difficulty functioning in typical childcare, home, and community settings. Analyses revealed some contradictory patterns that may be specific to this clinical sample. Children with higher exposure to ACE showed more biased social information processing patterns and their caregivers reported lower child social skills than caregivers of children with less exposure, however their inhibitory control levels were higher (better control) and staff reported that these children exhibited better social skills as well as better approaches to learning than children with less exposure. No such contradictions were found in relation to the caregiver’s exposure to ACE, as it was positively associated with a number of negative child and caregiver outcomes.

## 1. Introduction

Adverse childhood experiences (ACEs) are commonly defined as harmful experiences that occur in early childhood and a have a strong potential to traumatically affect the health and well-being of the individuals experiencing them [1]. They include economic hardship, physical, sexual, and emotional abuse, neglect, exposure to violence and criminality, and exposure to substance abuse and mental health problems in the household [1,2]. In childhood, exposure ACE has been found to have a profound negative impact on children’s health outcomes (e.g., [3,4,5,6]), and on children’s social and emotional outcomes (e.g., [3,6,7]). Yet, no study to date examined the effects of exposure to ACE in a clinical sample of preschoolers already showing multiple behavioral and emotional problems. It may be, however, that a study examining these associations in such a unique sample could have higher internal validity than large population-based samples, because non-ACE exposed children in that sample are more similar, behaviorally and emotionally, to the children exposed to ACE than in large population-based samples.

Accordingly, it is the aim of the present study to examine the associations among: (a) exposure to adverse childhood experiences of caregivers and children; and (b) children’s social, emotional, and academic adjustment, as well as their caregivers’ adjustment in a sample of preschool children enrolled in a specialized Therapeutic Nursery Program. Children were referred to this program because of significant developmental vulnerabilities associated with disruptions in their social, emotional, and behavioral regulation.

### 1.1. Exposure to Adverse Childhood Experiences

More than 50 percent of American adults and 46 percent of American children have experienced at least one ACE in their early years [1,8]. Moreover, in a comprehensive survey conducted by the U.S. Department of Justice, it was found that a large portion of the children for whom exposure to ACE was reported were exposed to particularly harmful early experiences, with more than 10 percent of all respondents suffering maltreatment, six percent experiencing sexual abuse, and 10 percent witnessing an adult family member acting violently against another family member [9]. Exposure to such harmful experiences of abuse and neglect in early childhood has been linked to a variety of negative social emotional outcomes such as insecure and disorganized attachment, lower self-esteem, poor relationships with peers, and maladjusted behavior in school [10,11,12,13,14]. Furthermore, the effects of maltreatment on social emotional development are long-lasting and remain a factor even after children are removed from the abusive or neglectful environment and placed in out-of-home care [15,16]. In general, exposure to adverse childhood experiences is considered to be the most persistent and change resistant environmental risk factor in early childhood, particularly when it involves 3–5 or more exposure types [1,5,7]. Whereas most research conducted on the effects of exposure to ACE focused on health outcomes of adults, some research was also conducted on the ways by which exposure to ACE affects children’s outcomes. The next section is a selective review of this research.

### 1.2. Exposure to ACE and Children’s Outcomes

Most ACE research relies on retrospective accounts of adults about their own exposure to ACE in childhood and the effects it has on their health and well-being as adults. Less research is available on the effects of exposure to adverse experiences while the individual is still in early childhood [7]. Most of the available research on these more concurrent effects has focused on the links between exposure to ACE and health problems in early childhood. Marie-Mitchell and O’Connor [6] reported that higher ACE scores among preschool children (ages 4 to 5) is associated with more injury-related visits in health centers, however, to less symptoms of asthma and to lower obesity. In contrast, a study of participants aged 0 to 20 found that higher levels of exposure to ACE were linked to higher rates of obesity [3]. A possible explanation for these contradictory findings is that the relationship between ACE and obesity changes over the years [6]. Moreover, exposure to 5 or more adversities was found to be related to more health complaints, to illnesses that required the assistance of a doctor, and to caregiver’s reports of somatic complaints of the child [5]. Finally, Flaherty and colleagues reported that in children ages 4 to 6, exposure to one adverse experience almost doubled the chances to develop overall poor health. Moreover, exposure to 4 or more adverse experiences almost tripled the likelihood for illness in children [4].

Less is known about the associations between exposure to ACE and children’s behavioral and psychological outcomes [7]. The available research about these associations suggests that exposure to ACE is related to behavior problems, developmental delays, and learning problems in early childhood [3,6], with exposure to three or more ACEs linked to below average language, literacy and math skills, attention problems, social problems and aggression [7].

In the present study, we aim to add to this research by examining the associations between exposure to ACE and preschool children’s cognitive and social outcomes. Moreover, to the best of our knowledge, the association between exposure to ACE (as measured with the ACES questionnaires) and social information processing has yet to be measured. But, as can be seen in the next section, SIP had been found to be highly associated with problem behavior as well as to exposure to violence in early childhood. Thus, we also examine in this study the associations between exposure to ACE and SIP.

### 1.3. Children’s Social Information Processing Patterns

The Social Information Processing model (SIP; [17,18]) emphasizes the covert mental mechanisms mediating an overt social stimulus and an overt social response. It describes a circular process in which five mental steps are activated in response to an external social cue and deactivated upon the individual’s enactment of a behavioral response. The five mental steps are: (1) encoding social cues; (2) interpreting the cue; (3) clarifying goals; (4) constructing a response; (5) making a decision on the response [17]. These five mental steps are followed by a sixth step: enacting the behavioral response. In this circular process, each mental step affects, and is affected by, a database of social behavior. This database includes the memory of past situations, acquired social rules, social schemes, and knowledge of appropriate and inappropriate social behaviors. If the individual’s database is dominated by harsh, unsafe, and unpredictable social experiences, as is often the case with children exposed to ACE, it is likely that social information processing may be distorted, and the knowledge of what is right or wrong, what is acceptable or unacceptable, and what is the correct response to a certain social situation may differ from normative social expectations [19].

In previous studies, processing of social information related to the peer group has been found to predict children’s behavior, especially conduct problems and maladjusted social behaviors. Numerous studies have found that children with distorted SIP behave more aggressively than children with non-distorted SIP (e.g., [20,21,22,23]), whereas other studies have found associations between distorted SIP and shy and withdrawn behaviors [24].

Studies examining the links between caregivers’ behaviors and SIP reported that negative parental behaviors, such as negative emotionality, criticism, and covert and overt hostility, were associated with children’s hostile attribution biases and aggressive tendencies in school (e.g., [25,26,27,28,29,30,31]). Especially important for the current examination, exposure to violence and abuse was also found to be a predictor of SIP. In studies examining these associations, it was found that young children exposed to such experiences early in their lives exhibited negative SIP biases (e.g., [23,25,32]).

With the exception of two recent studies [32,33], all studies reviewed here examined SIP in relation to hypothetical social interactions with peers, and, as of yet, no other studies have examined SIP patterns in relation to hypothetical interactions with the caregiver. In the current study, in addition to employing an interview assessing SIP of social interactions with peers, we also assess the SIP patterns of children when asked about hypothetical interactions with a caregiver.

This study is unique in examining the links between exposure to ACE and maladjusted behaviors and perceptions in a clinical sample of preschool children experiencing various behavioral, social, or emotional problems, which prevented their successful participation in regular preschool settings. These children were referred to a therapeutic nursery program, briefly discuss next.

### 1.4. Therapeutic Nursery Program (TNP)

The TNP in which the current study took place is a specialized family-focused, early childhood education-intervention program based in an early childhood mental health center. It was designed to improve the social and emotional development, as well as the educational outcomes of preschool age children (ages 3–5). Children are typically referred to the TNP to address a range of challenges related to disrupted social, emotional, and behavioral regulation. Referrals come from childcare providers, teachers, child welfare services, service providers, pediatricians, and caregivers. Children presenting externalizing problems often display safety concerns related to physical and verbal aggression that have led to difficulty functioning in typical daycare or preschool settings, as well as at home. Children may also present clinically significant internalizing problems as exemplified by anxious and depressive symptoms, somatic complaints, and isolating behaviors. At the most extreme, self-harming behaviors have also been evident. Regardless of their child’s difficulties, a common theme for most, if not all, caregivers, is feeling overwhelmed by their child’s distress and challenging behaviors, to the point of eroding the parent-child relationship. An underlying assumption of this TNP’s treatment model is that without comprehensive intervention, children referred to this program are at high risk for school failure, lifelong mental health complications, and strained family and peer relationships.

The families of the children in the TNP often face numerous external and internal stressors, which can include economic hardship, housing instability, lack of social support, domestic violence, parental substance abuse, parental mental illness, and intergenerational patterns of abuse, neglect, and other traumas. In addition, some of the children in TNP have experienced a change in primary caregiver such that relatives are caring for them or they are in foster care. Overall, most families have experienced major life disruptions during the past year (e.g., change in health of a family member, change in financial state of family, change in employment). Thus, the sample in this study could be described as a high-risk sample.

## 2. Materials and Methods

### 2.1. Participants and Procedure

Participants were 30 preschool children (21 boys, nine girls; mean age = 58.41 months; SD = 12.38; range 34 to 92 months) enrolled in the TNP, and their primary caregivers (19 mothers, four fathers, five grandmothers, and one other relative). Child’s race was reported by caregivers: 14 (48.3%) were African American, nine Latino/Hispanic (31%), five white (17.2%) and one child was identified as multiracial. Ten of the caregivers were never married (37%), seven were divorced or separated (25.9%), and ten were married (37%). Eleven (39.3%) caregivers had a college degree, whereas seven caregivers (25%) had a high-school diploma or did not complete high-school. 64.3% of caregivers worked either full (46.4%) or part (17.9%) time, and 20 caregivers (68.9%) reported a yearly income of less than $50,000 (11 of them reported on a yearly income of less than $25,000). All data collection activities took place within the center during normal operation hours. During intake, primary caregivers were asked to participate in the study. All caregivers of eligible children (i.e., not in foster care) signed their consent to participate in the study. Next, the primary caregiver was interviewed to obtain information about their own and their child’s exposure to ACEs and completed questionnaires about their child’s behavior, their relationship with the child and their locus of control. After these sessions, children’s SIP patterns as well as other outcomes were assessed via direct assessment. Finally, TNP staff completed questionnaires regarding the children’s social and academic adjustment in the program, and parent. The study’s protocol followed ethical guidelines and was approved by the Center’s Institutional Review Board (IRB) (Ethical Approval Code LC-2012-10).

### 2.2. Research Measures

#### 2.2.1. Questionnaires Completed by Caregivers

*Exposure to adverse childhood experiences* was assessed with the Adverse Childhood Experiences questionnaire (ACE; [1]). There are multiple ACE forms, two of which were used in the current study: (a) the original ACE—in which individuals report about their own adverse childhood experiences (until age 18)—was completed by the child’s primary caregiver; and (b) the Child ACE (CH-ACE)—in which the primary caregiver reports about the target child’s exposure to adverse childhood experiences since s/he was born. Each questionnaire includes 25 items related to physical, sexual and emotional abuse, exposure to violence, neglect, substance abuse in the household, household members’ mental health, arrests and incarcerations. The rating scales are different for different items. Some items are rated on a 4-point scale (0—Never, 1—Once, twice, 2—Sometimes, 3—Often, 4—Very often) while other items are YES or NO questions. A previous study reported good internal consistency (Cronbach Alpha) of 0.88 for the 10 binary categories created from the scales [2]. In the current study, Cronbach Alpha for the caregiver questionnaire was 0.77, and 0.82 for the child questionnaire.

*Child positive social behavior.* The child’s positive social behavior was assed using a 12-item questionnaire [34,35]. The items are drawn from the Personal Maturity Scale [36] and the Social Skills Rating System [37]. The scale is used to assess positive social behaviors such as sharing, helping, and complimenting others. The caregiver was asked to rate each item regarding the child’s behavior in the past month as 0 (“not true”); 1 (“somewhat true”); 2 (“very true”). The internal consistency score (Cronbach’s alpha) in previous research was 0.90 [23].

*Child behavior problems.* Child behavior problems were assessed using a 14-item questionnaire that includes four statements regarding aggressive behaviors (e.g., “hits or fights with others”), three statements regarding hyperactive behavior (e.g., “is very restless”) and seven statements regarding withdrawn behaviors (e.g., “keeps to himself or herself; tends to withdraw”). This scale is derived from the Personal Maturity Scale [36], the Child Behavior Checklist for Preschool-Age Children–Teacher Report [38], and the Behavior Problem Index [39]. Caregivers were asked to rate each statement as 0 (“not true”); 1 (“somewhat true”); 2 (“very true”). Internal consistency scores (Cronbach’s alpha) in previous research were 0.77 for withdrawn behaviors, 0.74 for hyperactive behaviors, and 0.85 for aggressive behaviors [40].

*Quality of relationship with the child.* The quality of the caregiver-child relationship was assessed using a measure that was partially derived from the child-parent relationship scale (CPRS; [41,42]). The 16-item questionnaire combines eight statements associated with the caregiver’s feelings of security/comfort in the relationship with the child (e.g., “I share an affectionate, warm relationship with my child”), and eight items associated with the caregiver’s feelings of anxiety/anger in the relationship (e.g., “my child and I always seem to be struggling with each other”). Each item is rated between 1 (“definitely does not apply”) to 4 (“definitely applies”). Internal consistency (Cronbach’s alpha) in previous research for the security scale was found to be 0.88 and for anxiety/anger scale, 0.87 [32].

*Locus of control* was measured using the self-mastery scale (SMS; [43]). This questionnaire is composed of seven items regarding locus of control, such as “there is really no way I can solve some of the problem I have” Two of the items (“I can do just about anything I really set my mind to do”; “what happens to me in the future depends mostly on me”) were reverse scored in order for a high score to represent an external locus of control. The items are rated between 0 (“strongly disagree”), to 3 (“strongly agree”). Cronbach’s alpha in previous research was found to be 0.75 [44].

#### 2.2.2. Child Assessment Measures

*Social Information Processing Interview, Preschool Version* (SIPI-P; [45])*.* This 20-min. structured interview is based on a storybook easel depicting a series of four vignettes in which a protagonist is either being excluded by two peers (the two *peer-exclusion* vignettes) or provoked by another peer (the two *peer-provocation* vignettes). The peers’ intent is portrayed as either ambiguous or non-hostile/accidental (never intentionally hostile).

**Figure A1:**
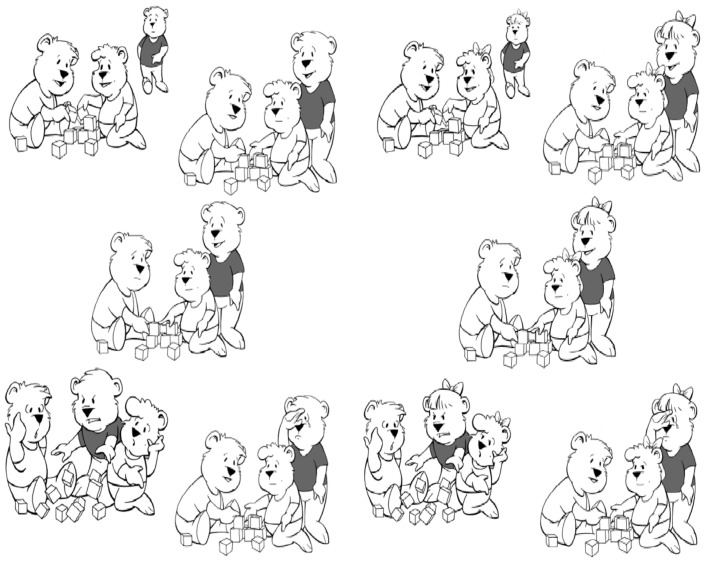
SIPI-P story: Peer entry example: Boys’ version on the left, girls’ version on the right—Story 1—Non-hostile exclusion. In the original measure, each picture appears on a separate page. Order of pictures: left to right, top to bottom.

The illustrations in the storybook are of cartoon bear characters and there are parallel picture books for boys and girls (see Appendix B, Figure A1. for an example of one vignette). As the child hears the story, the interviewer stops at scripted points and poses questions addressing the hypothesized information processing steps. Eight main scores are initially derived from the SIPI-P: (1) *efficient encoding* (α = 0.84), which is a summary score of the child responses to the question (asked once for each of four stories): “what happened in the story, from the beginning to the end” with higher scores representing better recollection; (2) *hostile attribution bias* (α = 0.69), which is a frequency count of the number of times the child describes the other child/ren as having hostile intents across the four stories (based on the question: “were the other child/ren mean or not mean?”). Thus, the range for this score is 0 to 4, with higher scores representing higher tendency to attribute hostile intent to peers; (3) *competent response construction*; (4) *aggressive response construction*; and (5) *inept response construction*. Each of these three scores represents a summary of the child’s responses to the question: “what would you do if this (whatever happened in the said story) happened to you?” The possible range of each of these scores is 0–4, with higher scores representing higher levels of competent/aggressive/inept response construction, respectively; (6) *competent response evaluation* (α = 0.87); (7) *aggressive response evaluation* (α = 0.80); and, (8) *inept response evaluation* (α = 0.86). Each of these three scores represents a summary of the children’s evaluation of a response (i.e., competent, aggressive, or inept) presented to them (e.g., the child is shown an aggressive response, for example, the child ruins the other children’s game, and is asked three questions: “was this a good thing or a bad thing to do?”; “if you had done this, will the other children love you?”; “if you had done this, will the other children let you play?). The possible range of each of these scores is 0–12 with higher scores representing higher levels of competent/aggressive/inept response evaluation, respectively.

*Social Information Processing—Parent-Child version* (SIPI-PC; [32]). This interview was used to evaluate the child’s responses to stories presenting interactions between a caregiver and a child. The interview contains five stories that are told in an order that reflects increasing levels of distress (from story 1: staying alone in the room, to story 5: getting lost in the mall). In the current study, the following variables were derived from this measure: (a) *response construction*—After hearing a story, children were asked what they would do or say if this had happened to them. Based on the child’s answers, three “response construction” scores were created: (1) competent/secure response construction (e.g., “I’ll ask for a band aid” in the “hurt knee” vignette); (2) aggressive/hostile response construction (e.g., I’ll tell her I don’t need her); and (3) avoidant response construction (e.g., I’ll do nothing). In each of the five stories the child’s response is coded for the three options. For example, if the child constructs a secure response in the first story, he is given “1” for competent code, and “0” for aggressive and for avoidant code. Because there are five stories, the three final scores range from 0 to 5. (b) *response evaluation*—after giving their answers, children were presented with three different possible responses to the portrayed scenario: competent, aggressive, and avoidant, and were asked what they think about each of the responses. These answers were used to create three “response evaluation” scores: (1) positive evaluation of a competent response; (2) positive evaluation of an aggressive response; and (3) positive evaluation of an avoidant response. The range of each of the three scores was 0–15 (3 questions in each story for every possible response. The child is given”0”—for negative evaluation, or “1”—for positive evaluation). The internal consistency reliability for response evaluations was 0.67 to 0.83 (Cronbach’s Alpha). (For more information about this measure, see Weisberger and Ziv, 2016 [32]).

*Child’s Inhibitory control.* Inhibitory control was measured with the Bear/Dragon test [46]. In this test, the experimenter showed the children a “nice” bear puppet (using a soft, high-pitched voice) and a “naughty” dragon puppet (using a gruff, low-pitched voice). Next, the experimenter explained to the children that in this game they need to follow what the bear asks them to do but never to follow the dragon’s requests. After practicing, there were 10 test trials with the bear (5 trials) and dragon (5 trials) commands in alternating order. Children were seated at a table throughout the task, and all actions involved hand movements. Performance on dragon trials was taken as an index of lack of self-control: 0 = no response; 1 = different response (e.g., the dragon says “touch your mouth” and the child touches her elbow); 2 = partial response (e.g., the dragon says “touch your nose” and the child is starting to move her hand towards her nose but stops in the middle of movement); 3 = full response. Thus, a higher score represents less inhibitory control and the possible score range for this test was 0 (no response on all 5 trials) to 15 (full response on all 5 trials).

*Children’s cognitive and academic capabilities* were measured with four different measures: (1) the Peabody Picture Vocabulary Test–Third Edition (PPVT-III; [47]) was used to assess children’s knowledge of word meanings by asking them to say or point to which of four pictures best shows the meaning of a word that is said aloud by the assessor. A series of words ranging from easy to difficult for children of a given age is presented, each accompanied by a picture plate consisting of four line drawings. For the current project, we used a shortened 48-item adaptive research version that was developed to be used in the FACES study [34]. (2) *Counting Blocks.* In order to assess early math skills, a one-to-one counting task was added to the assessment battery. The child was presented with a plate depicting two rows of 10 blocks (a total of 20 blocks) and asked to count them. The assessor marked the final number the child arrived at when he/she finished counting in one-to-one correspondence. This measure was previously used with Head Start children in the FACES study (e.g., [34]). (3) Woodcock-Johnson Psycho-Educational Battery-Revised. Two subscales of Woodcock-Johnson Revised (WJ-R; [48]) were included in the assessment battery: Letter-Word Identification and Applied Problems. The Letter-Word Identification subscale measures children’s reading skills by identifying isolated letters and words that appear in large type on the pages of the assessment booklet. The Applied Problems subscale measures children’s skills in analyzing and solving practical problems in early math. A stopping rule was applied for both scales (three consecutive items wrong). (4) Preschool CTOPP: Phonemic Awareness-Elision. The phonemic awareness task here is a shortened, adaptive version of the newly revised Preschool Children’s Test of Phonological Processing Elision task (Pre-CTOPP; [49]). This revised measure was first used and validated in the FACES study [34]. It is comprised from an Elision task that uses pictures to assist children in determining how the meaning of a word changes when one of its component sounds is taken away. The Pre-CTOPP task also has extensive practice items to help the children learn what is required of them.

#### 2.2.3. Questionnaires Completed by Classroom Staff (Main Teacher, Assistant Teacher, and Social Worker)

*Ratings of child behavior*. Staff were asked to rate three different types of behaviors: (1) positive social skills; (2) problem behaviors; and, (3) learning behaviors. *Positive social skills* were assessed using the competent social behavior scale [34,35]. The competent social behavior scale was created as part of the Head Start Family and Child Experiences Survey (FACES; [35]). There are 12 items in this measure, which were drawn from the Personal Maturity Scale [36] and the Social Skills Rating System [37]. It is composed of items dealing with helpful and compliant behaviors like “follows the teacher’s directions,” as well as items dealing with the child’s maturity and skill when interacting with other children, such as “invites others to join in activities.” All items are scored on a 3-point frequency scale ranging from “never” (coded 0) to “very often” (coded 2). The possible score range is 0–24. Internal consistency score (Cronbach’s Alpha) for this measure was 0.90.

*Problem behaviors.* This scale was also created as part of FACES and includes 14 items and was derived from the Personal Maturity Scale [36], the Child Behavior Checklist for Preschool-Age Children, the Teacher Report [38], and the Behavior Problem Index [39]. Teachers were asked to rate how often children exhibited various externalizing and internalizing behaviors: “never” (0), “sometimes” (1), or “very often” (2). An example of an externalizing item is “hits or fights with others.” An example of an internalizing item is “keeps to himself or herself; tends to withdraw.” In the present study, the two subscales (externalizing and internalizing) were highly correlated (*r* = 0.65) and thus were combined into one “behavior problems” scale with a possible range of 0–28. Internal consistency score (Cronbach’s Alpha) for this scale was 0.84.

*Learning behaviors* were assessed with the Preschool Learning Behavior Scale (PLBS; [50]). The PLBS is a 29-item rating scale of learning behaviors within the classroom; each item is rated on a 3-point scale: (1) “most often applies,” (2) “sometimes applies,” (3) “or doesn’t apply.” Three dimensions found to represent coherent factors in previous research (e.g., [51]) are derived from the PLBS: competence motivation, attention/persistence, and attitude toward learning. The competence motivation scale assesses children’s willingness to take on tasks and their determination for completing them successfully (e.g., “reluctant to tackle a new activity”). The attention/persistence dimension measures the degree to which children pay attention and are able to persist with difficult tasks (e.g., “tries hard, but concentration soon fades and performance deteriorates”). The attitude toward learning dimension focuses on such concepts as children’s willingness to be helped, desire to please the teacher, and ability to cope when frustrated (e.g., “doesn’t achieve anything constructive when in a sulky mood”). Internal consistency scores (Cronbach’s Alphas) for competence motivation, attention/persistence, and attitude toward learning scales in this study were 0.90, 0.89, and 0.87, respectively.

## 3. Results

### 3.1. Descriptive Statistics and Preliminary Analysis

Descriptive statistics (mean, standard deviation, possible range, observed range, and skewness) for all examined variables are presented in Table A1. (Appendix A). We first wanted to examine whether child’s gender and age are associated with any of the outcome variables. Thus, a series of *t*-tests were used to examine gender differences and zero-order Pearson correlations were used to examine associations between age and the outcome variables. There were no gender differences in any of the examined outcomes, but age was significantly associated with a large number of outcome variables and thus was entered as a control variable in the main analysis whenever relevant. We also wanted to examine the association between the child’s and caregiver’s exposure to ACE. This association was significant, but not as strong as might have been expected: *r* (29) = 0.33, *p* = 0.043 (1-tailed).

### 3.2. Main Analysis

#### 3.2.1. Analytical Approach

To examine the association between the level of child’s and caregiver’s exposure to ACE and the various outcomes, we conducted a series of bivariate (in cases where age was not associated with the examined outcome) or partial (in cases were age was related to the measured outcome), Pearson correlation. In cases where the correlations found were in accordance with our initial hypotheses, we report significance level based on a 1-tailed test. When findings were contradictory to our initial hypotheses, we report significance level based on a 2-tailed test.

#### 3.2.2. Child Exposure to ACE and Children’s Outcomes

We examined the associations between the child’s exposure to ACE (as reported by the caregiver) and various children’s outcomes: Academic (the teacher reported approaches to learning, and the child assessment cognitive measures), and social emotional (child’s SIP and inhibition, teacher and caregiver reported social behaviors).

*Academic and cognitive outcomes*: There were no significant associations between the child’s exposure to ACE and any of the child’s cognitive assessments (PPVT, WJ-III, counting blocks, and the CTOPP). On the other hand, significant associations emerged between the main teacher’s and the assistant teacher’s reports on the child’s PLBS *Attitude toward learning* score, but in the opposite direction then expected: both teachers reported that children with higher exposure to ACE have shown better attitude towards learning: *r* (26) = −0.36, *p* = 0.075 (2-tailed); and *r* (26) = −0.39, *p* = 0.050 (2-tailed), respectively.

*Social emotional outcomes.* The child’s level of exposure to ACE was significantly associated with a number of social and emotional outcomes. First, higher levels of exposure to ACE was positively associated with two negatively termed SIP variables: Hostile attribution, *r* (28) = 0.38, *p* = 0.031 (1-tailed, partial correlation controlling for age), and, aggressive response generation, *r* (28) = 0.36, *p* = 0.041 (1-tailed). Higher exposure to ACE was also positively associated with more problem behavior, as reported by the caregiver: *r* (28) = 0.36, *p* = 0.031 (1-tailed). Finally, higher exposure to ACE was also positively associated with the caregiver’s report of feelings of anxiety/anger in the relationship with the child: *r* (28) = 0.52, *p* = 0.002 (1-tailed).

On the other hand, again, higher exposure to ACE was associated with better social outcomes as reported by staff: both the main teacher and the social worker reported on higher social skills for children with higher exposure levels, *r* (26) = 0.41, *p* = 0.039 (2-tailed); and *r* (25) = 0.37, *p* = 0.097 (2-tailed), respectively. In addition, children who were reported to be more exposed to ACE have shown higher levels of inhibitory control: *r* (28) = −0.41, *p* = 0.040 (2-tailed, controlling for age).

#### 3.2.3. Caregiver Exposure to ACE and Child and Caregiver Outcomes

Caregiver’s exposure to ACE was not significantly associated with any of the child assessment outcomes and to only two outcome reported by the TNP staff: it was negatively associated with the child’s attitude towards learning, *r* (28) = 0.61, *p* = 0.002 (1-tailed, controlling for age); and with the child’s persistence, *r* (28) = 0.42, *p* = 0.030 (1-tailed, controlling for age), both as reported by the classroom social workers. The caregiver’s level of exposure to ACE was also associated with a number of outcomes as reported by the caregiver: it was positively associated with the child’s problem behavior, *r* (28) = 0.40, *p* = 0.018 (1-tailed); with the caregiver’s report of feelings of anxiety/anger in the relationship with the child: *r* (28) = 0.34, *p* = 0.041 (1-tailed); and with the caregiver’s feeling of less locus of control: *r* (28) = 0.54, *p* = 0.002 (1-tailed).

## 4. Discussion

This study was conducted in order to examine the associations between children and caregivers’ exposure to ACE and children and caregivers’ multiple outcomes, in particular, social-emotional outcomes. Whereas a small number of studies previously examined the associations between exposure to ACE and socioemotional outcomes in children (e.g., [3,6,7]), the present study is different in that the examination took place in a clinical sample of children that are already exhibiting a host of socioemotional difficulties. Indeed, our findings were somewhat different from these previous studies as significant links between exposure to ACE and children and caregivers’ outcomes were found, however, the pattern of association was unexpected and, in some cases, even contradictory to initial expectations, suggesting that in some contexts, exposure to ACE may have surprising effects that are not typically hypothesized. These findings have important theoretical implications as well as significant implications to the clinical and educational work within these settings. These implications are discussed below.

### 4.1. Theoretical Implications

The research field examining the associations between exposure to adverse childhood experiences and the general population of caregiver’ and child outcomes is already quite established. However, our findings add to this body of research a type of refinement in distinguishing between the effects of caregivers’ and children’s exposure in this particular sample of high risk families. There were no surprises here in terms of the associations between caregivers’ exposure to ACE and children’s and, particularly, caregivers’ outcomes. Our findings indicate that caregivers who were exposed to adverse experiences as children suffer lasting impacts in that when they become parents themselves, they are more likely to feel that their relationship with the child is going the wrong way (i.e., by describing high levels of anger and anxiety in these relationships), and are more critical of their child’s behavior than caregivers who experienced less adversity. A particularly strong association was found between the caregiver exposure to ACE and his/her locus of control. This strong positive association suggests that caregivers with higher levels of exposure feel they possess less control over their lives. These findings are in line with previous findings in adult population that found that higher levels of exposure to ACE are associated with poorer psychological outcomes such as higher levels of depression, anxiety, less control, and lower life satisfaction (e.g., [52]).

Findings relating to the outcomes of the child’s exposure to ACE, were more complicated, however. On the one hand, we found expected positive associations between exposure to ACE and child SIP biases, with more exposure associated with higher levels of hostile attribution bias and aggressive response generation. Also as expected, child’s higher exposure to ACE was positively associated with more problem behavior (as reported by the caregiver) and higher levels of insecurity/anxiety in the relationship with the caregiver (also as reported by the caregiver). These two latter positive associations are similar to those reported for the caregiver’s exposure to ACE but are likely quite independent from each other as the direct correlation between the child’s and caregiver’s exposure to ACE was smaller than expected (*r* = 0.33).

On the other hand, unexpected associations were found between the staff report of the child’s behavior and his/her exposure to ACE. First, higher exposure to ACE was associated with better social outcomes as reported by staff: both the main teacher and the social worker reported on higher social skills for children with higher exposure levels. Second, significant unexpected associations were also found between exposure to ACE and academic outcomes in both the main teacher and the assistant teacher reports. Both teachers reported that children with higher exposure to ACE have shown better attitude towards learning. In addition, one similar unexpected association between ACE and children’s outcomes was found in one of our direct assessment: children who were reported to be more exposed to ACE have shown higher levels of inhibitory control (i.e., performed better on the bear-dragon test).

The differences between teachers and caregivers’ reports on problem behavior are well established in different populations (e.g., [53,54,55,56]) with caregivers usually reporting higher levels of problem behavior than teachers. However, to the best of our knowledge, no study to date has reported completely opposite associations as found here. Based on these reports, it could be that within this particular clinical sample, there is a bigger difference between the behavior in the home and in the classroom in children with higher levels of exposure to ACE than in children with lower exposure. There could be a number of different explanations to these contradictory findings. First, as part of the TNP model of care, all staff are part of the evaluation process and learn about the child’s and caregiver/family’s history at intake and throughout the families’ participation. They are also involved in crisis management and in helping caregivers improve their own regulation as well as that of their children. Thus, they continuously learn about the family’s traumas as the relationship deepens over the year or two. Within this context, it is possible that because of their deep knowledge of the family, they may be particularly sensitive towards the exposed children. Second, it is entirely possible that exposure to ACE is indeed associated with better social behavior in the classroom but not at the home. This is also supported by the finding that higher levels of exposure to ACE were positively associated with better inhibitory control. The ability to self-control is an important social skill as well as an academic goal in the TNP, thus this finding seems to converge well with the staff report. Why does this ability not seem to be reflected in the child’s behavior at home? Perhaps because this is likely the place where the exposure occurred or because caregivers look at other types of behaviors (e.g., relationships with siblings), or the less structured environment in the home in contrast to the TNP with its therapeutic milieu and 3:1 trained staff to child ratio.

Still, there is likely more to these contradictions because these explanations do not cover another important finding in this study that more exposure to ACE is associated with more biased social information processing patterns. If indeed the explanation is so simple—children behave differently at home and at school—why are their SIP patterns still biased? One explanation is that exposure to ACE in this clinical sample affects differently children’s mental representations and their manifested behavior. In most samples, these associations are quite straightforward: more exposure is associated with more problem behavior *as well as* more SIP biases. Perhaps these straightforward associations do not work that well in this sample because *all* children already exhibit multiple behavioral issues but the reasons for these behaviors may be different. In some, the behaviors are indeed associated with their early experiences, but in others, they may be related to more constitutional and/or medical antecedents. Perhaps with the ACE exposed children, it is more effective to facilitate behavior change through the relational-based, experiential learning in the class, whereas for the other children that manifest behavior problems which are likely not the result of early exposure to ACE, the route to change may rely on a different route.

### 4.2. Clinical Implications

There are important clinical implications to these findings as well. First, our findings about the discrepancies between mental representations of social interactions (i.e., SIP) and social behavior in children exposed to ACE are extremely important information for early intervention programs. Typically, if staff see improvements in social behavior and inhibitory control, the logical assumption is that their individualized treatment plan is working well. While this is likely true, these findings suggest that there is another layer that needs to be explored. If this information is supplemented by the additional layer of knowledge that the child still holds mental biases that are manifested, for example, in his/her perception of others as hostile towards him or her (as was found here), the conclusion may be very different. This may help explain some of the differences between effectiveness in short-term versus long-term intervention models. Thus, an important implication for clinical staff could be that for a more complete evaluation of the child’s social emotional situation, it may not be enough to observe his or her explicit behavior. They must also assess the child’s mental representations of self and others. Second, if our above speculations that changes in social behavior in the program are related to the initial reasons for the maladjustments that brought these children to the TNP are true, it might mean that the treatment in the TNP may hold different levels of effectiveness as a function of these reasons. This information is important as it emphasize even more the need for individual care plan that is tailored based not only on the initial assessment of the child in intake but also on complete as possible information on the history of the child’s experiences before arriving at the program. For example, two children may show very similar behaviors during intake but the effectiveness of treatment may differ as a function of their history.

Finally, our findings regarding the effects of ACE on caregivers, as well as their more negative views of their children, highlights the need for comprehensive supports for caregivers’ who, in addition to their own challenges, are also faced with their own children’s difficulties with social, emotional, and behavioral regulation as manifested in their placement in the TNP.

### 4.3. Study Limitations and Future Directions

There are a number of limitations that should be noted and addressed in future studies. First, the sample is small, and thus the risk of chance findings is enhanced. Because of the small sample, it was not possible to use more sophisticated analyses on these data, for example, to examine the possible moderating effects of the caregiver’s perceptions and behaviors on the link between exposure to ACE and children perceptions and behaviors, or, to use multivariate analyses to examine the possible confounding effects of other variables.

Second, as a study that took place in a center that works from an attachment theory perspective, there are two important measurement limitations. First, the study did not include a direct assessment of the child’s attachment security. Second, the study did not include an assessment of the caregiver’s attachment representations. Future studies examining links between exposure to ACE, caregivers’ outcomes, and children’s perceptions and behaviors, may consider including measures of the caregiver’s attachment representation (such as the Adult Attachment Interview, AAI; [57,58]), and of the child’s attachment security (such as the modified Strange Situation Procedure for preschoolers; [59]). The inclusion of such measures of attachment could provide a more complete understanding of the links found here. For example, if, and how, the caregiver’s representations of self are associated with his/her representations of the child; whether his/her representations of self are associated with the child’s perceptions and behaviors, directly or indirectly; and, whether these expected associations are evident in families challenged with environmental risk factors.

## 5. Conclusions

The current study is unique in examining the links between exposure to ACE and maladjusted behaviors and perceptions in a sample of preschool children that had already been diagnosed with various conduct disorders that prevents their participation in regular preschool setting.

Analyses showed some contradicting patterns that are likely specific to this clinical sample. Whereas children with higher exposure to ACE showed more biased social information processing patterns and their caregivers reported lower social skills than caregivers of children with less exposure, their inhibitory control levels were higher (better control) and staff reported that these children exhibit better social skills as well as better approaches to learning than children with less exposure. No such contradictions were found in relation to the caregiver’s exposure to ACE, as it was positively associated with a number of negative child and caregiver outcomes. The unique pattern of findings reported here bear important theoretical and clinical implications that are likely to advance the field. However, the unexpected contradictory findings described above suggest the need for additional studies and a general carefulness in reaching any overarching conclusions about these findings.

## Appendix A

**Table A1:** Descriptive statistics for all study variables.

Skewness	Observed Range	Possible Range	SD	M	Variable Name
1.08	0–32	0–57	8.39	9.79	ACE-Caregiver
0.91	0–11	0–33	3.13	3	ACE-Child
					Caregiver report
0.04	5–14	0–24	2.51	9.18	Positive social skills
0.79	3–23	0–28	4.81	10.79	Behavior problems
−0.79	22–32	0–32	2.49	28.25	Quality of relationship with the child
0.44	0–15	0–21	3.79	5.71	Caregiver Locus of control
					Child assessment
1.63	0–11	0–13	3.46	2.10	SIP-efficient encoding
−0.06	0–4	0–4	1.53	2.07	SIP-hostile attribution
0.90	0–4	0–4	1.46	1.13	SIP-competent response construction
1.36	0–4	0–4	1.26	0.93	SIP-aggressive response construction
4.34	0–4	0–4	0.77	0.23	SIP-inept response construction
−1.34	1–12	0–12	3.31	9.21	SIP-competent response evaluation
0.97	0–10	0–12	3.19	2.76	SIP-aggressive response evaluation
0.59	0–10	0–12	2.65	3.62	SIP-inept response evaluation
0.12	0–5	0–5	1.65	1.90	SIP-PC-competent response construction
2.88	0–3	0–5	0.7	0.28	SIP-PC-aggressive response construction
2.73	0–5	0–5	1.15	0.52	SIP-PC-inept response construction
−0.72	6–15	0–15	2.89	12.24	SIP-PC-competent response evaluation
0.59	0–12	0–15	3.78	3.97	SIP-PC-aggressive response evaluation
0.74	0–15	0–15	3.62	5.90	SIP-PC-inept response evaluation
0.47	0–15	0–15	7.12	5.90	Inhibitory control
					Staff ratings
−0.13	9–21	0–24	3.20	15.08	Positive social skills (main teacher)
−0.67	5–21	0–28	4.72	14.42	Problem behaviors (main teacher)
0.3	0–17	0–22	4.11	8.31	Learning behavior—motivation (main teacher)
−0.80	0–13	0–18	3.96	8.58	Learning behavior—persistent (main teacher)
−0.80	1–9	0–14	2.30	5.81	Learning behavior—attitude (main teacher)
0.27	7–22	0–24	4.28	14.12	Positive social skills (assistant teacher)
0.1	4–20	0–28	4.8	11.69	Problem behaviors (assistant teacher)
1.57	0–15	0–22	3.19	4.73	Learning behavior—motivation (assistant teacher)
−0.41	0–12	0–18	3.62	6.73	Learning behavior—persistent (assistant teacher)
−0.16	1–9	0–14	2.63	5.04	Learning behavior—attitude (assistant teacher)
0.30	7–20	0–24	3.86	13	Positive social skills (social worker)
−0.29	2–20	0–28	4.66	11.95	Problem behaviors (social worker)
0.78	0–16	0–22	5.17	5.24	Learning behavior—motivation (social worker)
0.12	0–14	0–18	3.88	6.10	Learning behavior—persistent (social worker)
−0.27	1–9	0–14	2.52	4.48	Learning behavior—attitude (social worker)
